# Short-Term Outcomes and Cost of Robotic Versus Open Distal Pancreatectomy During the Initial Phase of a Robotic Pancreatectomy Program

**DOI:** 10.1097/AS9.0000000000000687

**Published:** 2026-07-02

**Authors:** Anneliese N. Hierl, Jessica E. Maxwell, Laura R. Prakash, Yi-Ju Chiang, Jeeva Ajith, Monica D. Garza, Rebecca A. Snyder, Michael P. Kim, Hop S. Tran Cao, Ching-Wei D. Tzeng, Jeffrey E. Lee, Matthew H. G. Katz, Naruhiko Ikoma

**Affiliations:** From the * Department of Surgical Oncology, The University of Texas MD Anderson Cancer Center, Houston, TX; † Department of Finance and Analytics, The University of Texas MD Anderson Cancer Center, Houston, TX.

**Keywords:** robotic distal pancreatectomy, open distal pancreatectomy, costs, short-term outcomes, opioid use

## Abstract

**Background:**

This retrospective study evaluates the safety and cost-effectiveness of robotic distal pancreatectomy (RDP) during surgeons’ early experience, a period for which data remain limited. We compared outcomes and costs of RDP versus open distal pancreatectomy (ODP) during the initial phase of our robotic program.

**Methods:**

RDP and ODP cases from January 2018 to May 2024 were analyzed using a prospective database to obtain clinicopathologic and 90-day outcomes. Costs were expressed as ratios to ODP operative cost. 1:1 propensity-matched cohorts were compared.

**Results:**

One hundred nineteen RDP and 164 ODP cases were identified; matching yielded 101 per group. Major complications (Accordion grade ≥3: 10% vs 17%; *P* = 0.15), 90-day readmissions (17% vs 17%; *P* > 0.99), and operative time (266 vs 268 minutes; *P* = 0.95) were similar. RDP had higher rates of biochemical leak (34% vs 20%; *P* = 0.026) but similar rates of grade B postoperative pancreatic fistula (13% vs 21%; *P* = 0.13). RDP had higher intraoperative (1.25 vs 1.00) but lower index stay (2.48 vs 2.99) and 30-day (2.73 vs 3.41) cost ratios, all *P* < 0.001. RDP had lower inpatient opioid use (36 vs 111 mg; *P* < 0.001) and shorter hospital stay (3 vs 5 days; *P* < 0.001).

**Conclusions:**

RDP was safe, with comparable 90-day outcomes and lower 30-day costs compared to ODP during RDP program implementation.

## INTRODUCTION

Minimally invasive pancreatic surgery has gained popularity over the past 2 decades because of advancements in robotic surgery.^1–3^ The LEOPARD randomized control trial and subsequent meta-analyses have demonstrated that minimally invasive distal pancreatectomy is associated with lower estimated blood loss (EBL) and shorter hospital stays than open distal pancreatectomy (ODP).^3–5^ Among minimally invasive approaches, robotic distal pancreatectomy (RDP) has emerged as a promising technique. Compared with laparoscopic surgery, it offers advantages including enhanced dexterity, 3D visualization, increased rates of spleen preservation, and improved surgical outcomes.^6–8^

Although prior studies have demonstrated favorable outcomes with RDP, most studies reflect experiences at high-volume centers or from surgeons beyond the learning phase, which is often defined as the first 20 to 50 cases.^9,10^ As a result, limited data are available on perioperative outcomes and healthcare costs during the initial implementation phase of a robotic pancreatectomy program. We began our robotic pancreatectomy program in late 2018 and have reported our early experience. As the RDP program expanded to include 6 surgeons, we maintained favorable safety outcomes and observed a progressive reduction in operative time over successive cases, reflecting the learning curve associated with RDP.^11^ However, those analyses did not include comprehensive value-based metrics, such as perioperative costs or opioid use, or comparisons with patients undergoing ODP.

Globally, a major concern regarding the adoption of robotic surgery is its high upfront cost, which includes the robotic platform, specialized instruments, ongoing maintenance, and longer operative times that contribute to increased operating room and labor expenses.^12^ Additionally, it is well established that postoperative complications—particularly those related to pancreatic fistula—significantly increase total hospital costs.^13^ Cost can be considered an aggregate indicator that correlates with multiple aspects of surgical care, including case complexity, resource utilization, and the demands of postoperative management. While RDP may be associated with lower overall costs than ODP after surgeons pass the initial learning curve,^14^ this is contingent on reductions in the complication rates and lengths of stay. However, during the early phase of adoption, immature robotic surgical skills may lead to higher complication rates and associated costs.^15^ Similarly, although robotic surgery has been linked to reduced postoperative opioid requirements,^16^ these potential benefits have not been specifically evaluated during the early implementation phase of RDP.

To address this knowledge gap as well as to confirm safe and cost-effective implementation of our RDP program, we aimed to compare the short-term postoperative outcomes, including surgical outcomes, perioperative costs, and opioid use, of RDP and ODP during the implementation phase of our institution’s robotic pancreatectomy program by using a propensity score-matching (PSM) approach. We hypothesized that, compared with ODP, RDP would not compromise 90-day outcomes. In addition, RDP might increase the intraoperative cost, but the total perioperative costs would be mitigated by shorter lengths of stay and result in lower opioid use, even during the launch of our robotic pancreatectomy program.

## METHODS

### Study Design

This was a single-center retrospective analysis of all consecutive patients undergoing RDP or ODP from January 2018 (the year we started our robotic pancreatectomy program) through May 2024 at our tertiary cancer center. Patients who underwent concurrent resections of other organs, except for splenectomies and cholecystectomies, were excluded. Laparoscopic distal pancreatectomy was not routinely performed at our institution during the study period and, therefore, was not included in the analysis. This study was approved by our Institutional Review Board (PA18-0854). The need for patient consent was waived.

### Perioperative Management

All cases were managed according to our standardized, risk-stratified pancreatectomy pathways, which were implemented prior to the study period and applied uniformly to both robotic and ODP patients. These pathways include standardized drain management, opioid-sparing, multimodal pain management protocols, and discharge opioid prescriptions based on a 5× multiplier algorithm.^17–19^ The method of intraoperative pancreatic stump closure was at the discretion of the operating surgeon and was not standardized across cases.

This study included our initial cases of RDP. Most were performed primarily or collaboratively by a lead surgeon in our robotic pancreatectomy program, along with 5 other experienced surgical oncologists.^12^ RDP is a relatively straightforward operation without reconstruction; thus, it was included in the initial phase of our robotic surgery program. To maximize synergetic learning of the relevant robotic surgical oncology skills, our team performed various operations, including pancreatectomies and gastrectomies.^20^

### Surgical Outcomes

Data collected included demographic characteristics, body mass index, tumor histologic type and pathologic information, American Society of Anesthesiology score, operation duration, EBL, postoperative blood transfusions, and length of hospital stay for the index admission. Laboratory tests, including white blood cell (WBC) counts and drain fluid amylase (DFA) levels, were collected.

Clinicopathologic data and 90-day postoperative outcomes were extracted from a prospectively maintained departmental pancreatectomy database. This database is updated biweekly by a multidisciplinary team of surgeons and advanced practice providers to capture the incidence and nature of complications within 90 days of surgery.^21^ Postoperative complications were graded using the Accordion Severity Grading System^22^ and defined according to International Study Group of Pancreatic Surgery (ISGPS) criteria.^23–25^ These criteria included pancreatic fistula, delayed gastric emptying, cardiac and pulmonary events, thromboembolism, infections, readmissions, and mortality. Data collection was supplemented by retrospective review of the electronic health record (Epic Systems, Verona, WI).

### Cost Comparisons

All financial data used in this study were collected from the MD Anderson Finance and Analytics Department. The total hospital cost was defined as the estimated total cost to the hospital for providing a particular service.^26^ The 30-day period was used to exclude adjuvant chemotherapy. We calculated the costs of intraoperative care (operating room costs, including procedure and anesthesia), postoperative inpatient care, readmission, and outpatient care within 30 days after surgery. The total index stay cost was defined as the sum of intraoperative and postoperative inpatient care costs, whereas the postdischarge cost was defined as the sum of the readmission and outpatient care costs. The total perioperative cost was the aggregate of all these costs.

Costs were categorized following the methodology used in our previous studies.^12^ The operating room cost included expenses related to the use of the operating room (room use time, from patient entry to exit), the costs of instruments (including initiation and maintenance of instruments, such as surgical robots, appropriately accounted for with each individual use), radiology imaging costs when such imaging was performed in the operating room (added according to the modality used and the duration of use), and labor costs. Labor costs included nurses and respiratory, physical, occupational, and speech and language therapists. Professional fees for surgeons or anesthesiologists were not included, as we could not reliably and consistently estimate their procedure costs due to considerable variation in physicians’ salaries and the absence of Current Procedural Terminology codes for robotic surgeries. Costs specific to the robotic surgical system (e.g., lease, depreciation, and maintenance) were updated annually and added under revenue code 360. Costs for “laboratory” and “pharmacy” represented the total costs incurred throughout the entire hospitalization, including both the surgery and the remaining hospital stay. Surgical costs included expenses for any reoperations during the same hospitalization, as these were challenging to separate.

All cost data were converted to ratios relative to the mean intraoperative cost of ODP during the study period, allowing for cost representation without disclosing proprietary financial information.

### Opioid Outcomes

Different types and administering routes of opioid volumes were converted into milligrams of oral morphine equivalents (OMEs) using a hospital-approved and previously published conversion ratio.^27^ Preoperative (in preoperative holding area), intraoperative, and postoperative OMEs were calculated separately and summed for each hospital day. All patients received opioid-sparing, multimodal pain regimens (ie, routine use of acetaminophen, nonsteroidal antiinflammatory drugs, and muscle relaxers) according to our clinical pathways.^18^ Most patients underwent a transverse abdominis plane block under ultrasound or laparoscopic guidance.^28^ The amount of opioid injected via an epidural catheter was not included in our opioid outcomes due to the difficulty in confirming the analgesic volume.

### Statistical Analysis and PSM

PSM was conducted to create comparable cohorts of patients undergoing RDP and ODP while minimizing baseline differences in patient and disease characteristics inherent to retrospective comparisons. Propensity scores were calculated for all patients using the following covariates: age, body mass index, American Society of Anesthesiology score, and tumor histologic type (including pancreatic ductal adenocarcinoma, pancreatic neuroendocrine tumor, cyst, other [comprised of leiomyosarcoma, renal primary tumor, colorectal primary tumor, sarcoma, and solid pseudopapillary tumors]). Matching was performed 1:1 using nearest-neighbor matching with a caliper width of 0.25 standard deviations and exact matching on sex. Covariate balance before and after matching was assessed using absolute standardized differences. An absolute standard difference of <0.1 was considered indicative of acceptable balance between the RDP and ODP groups (Table 1).

**TABLE 1.** Patients’ Clinicopathologic Characteristics

	Unmatched Cohort					Matched Cohort				
		Approach					Approach			
Characteristic	Overall (N = 283)	Open (N = 164)	Robotic (N = 119)	*P* *	Stdiff	Overall (N = 202)	Open (N = 101)	Robotic (N = 101)	*P* *	Stdiff
Median age, years (IQR)	65 (55–71)	66 (57–72)	62 (53–70)	0.067	0.190	64 (55–71)	65 (57–72)	63 (54–69)	0.15	0.170
Sex				0.21	0.152				>0.99	<0.001
Male	135 (48)	73 (45)	62 (52)			108 (53)	54 (53)	54 (53)		
Female	148 (52)	91 (55)	57 (48)			94 (47)	47 (47)	47 (47)		
Race and ethnicity				0.54	0.226				0.17	0.372
White or Caucasian	200 (71)	121 (74)	79 (66)			142 (70)	76 (75)	66 (65)		
Hispanic or Latino	33 (12)	17 (10)	16 (13)			24 (12)	9 (8.9)	15 (15)		
Asian	27 (10)	15 (9)	12 (10)			18 (9)	7 (7)	11 (11)		
Black or African American	19 (7)	10 (6)	9 (8)			15 (7)	9 (9)	6 (6)		
Other	4 (1)	1 (1)	3 (3)			3 (2)	0 (0)	3 (3)		
Median BMI, kg/m^2^ (IQR)	27.6 (23.9–32.1)	26.8 (23.7–30.7)	28.6 (25.0–33.1)	0.003	0.382	28.2 (24.4–32.1)	27.3 (24.2–31.0)	28.4 (25.0–32.4)	0.16	0.235
ASA score				0.015	0.341				>0.99	0.266
Class 2	15 (5)	4 (2)	11 (9)			6 (3)	3 (3)	3 (3)		
Class 3	267 (94)	159 (97)	108 (91)			196 (97)	98 (97)	98 (97)		
Class 4	1 (1)	1 (1)	0 (0)			0 (0)	0 (0)	0 (0)		
Smoking status				0.31	0.194				0.16	0.266
Current	8 (3)	4 (2)	4 (3)			6 (3)	2 (2)	4 (4)		
Former	102 (36)	65 (40)	37 (31)			72 (36)	42 (42)	30 (30)		
Never	173 (61)	95 (58)	78 (66)			124 (61)	57 (56)	67 (66)		
Histologic type				<0.001	0.657				0.72	0.165
PNET	80 (28)	40 (24)	40 (34)			71 (35)	33 (33)	38 (38)		
PDAC	114 (40)	85 (52)	29 (24)			65 (32)	36 (36)	29 (29)		
Cyst	64 (23)	24 (15)	40 (34)			65 (32)	23 (23)	26 (26)		
Other	25 (9)	15 (9)	10 (8)			17 (8)	9 (8)	8 (8)		

All values are n (%) unless otherwise indicated.

* Wilcoxon rank-sum test; Pearson chi-squared test; Fisher exact test.

ASA indicates American Society of Anesthesiology; BMI, body mass index; IQR, interquartile range; PDAC, pancreatic ductal adenocarcinoma; PNET, pancreatic neuroendocrine tumor; other, including metastasis and solid pseudopapillary tumor; Stdiff, standard difference.

The primary outcome of this study was the total 30-day perioperative cost (ie, the best approximation of the hospital’s expenditure for the provided services during both the initial hospitalization and the subsequent 30 days of postoperative care). Variability in costs was assessed using Levene test for equality of variances. Other surgical outcomes, including opioid consumption, were summarized and compared between RDP and ODP. Categorical variables were described as counts and percentages and compared using chi-squared and Fisher exact tests for unmatched data, and McNemar test was used for matched data. Continuous variables were described as medians and interquartile ranges and compared using the Mann–Whitney *U* test in unmatched data, while the Wilcoxon signed-rank test was applied to matched continuous data. To evaluate the independent association between surgical approach and 30-day cost, a multivariable linear regression analysis was performed in the propensity score-matched cohort. Covariates included clinically relevant factors and variables associated with cost on univariable analysis. The alpha level was defined as *P* < 0.05, and all tests were 2-sided. All statistical analyses were performed using SAS Enterprise Guide 7.15 (SAS Inc., Cary, NC).

## RESULTS

### Patient Characteristics

We identified and included 283 patients, of whom 119 underwent RDP and 164 underwent ODP. The annual RDP case volume ranged from 1 to 32, with 1 case performed in 2018 due to the robotic program launching in October of that year, and the case volume increased steadily over the study period.^12^ No patients needed conversion to open laparotomy. After PSM, 202 patients were included, with 101 patients in each group. The matched groups had no significant differences in patient or disease characteristics (Table 1). After matching, the median age was 64 years (interquartile range: 55–71) and the median body mass index was 28.2 kg/m^2^ (interquartile range: 24.4–32.1 kg/m^2^).

### Cost Comparisons

**FIGURE 1.**
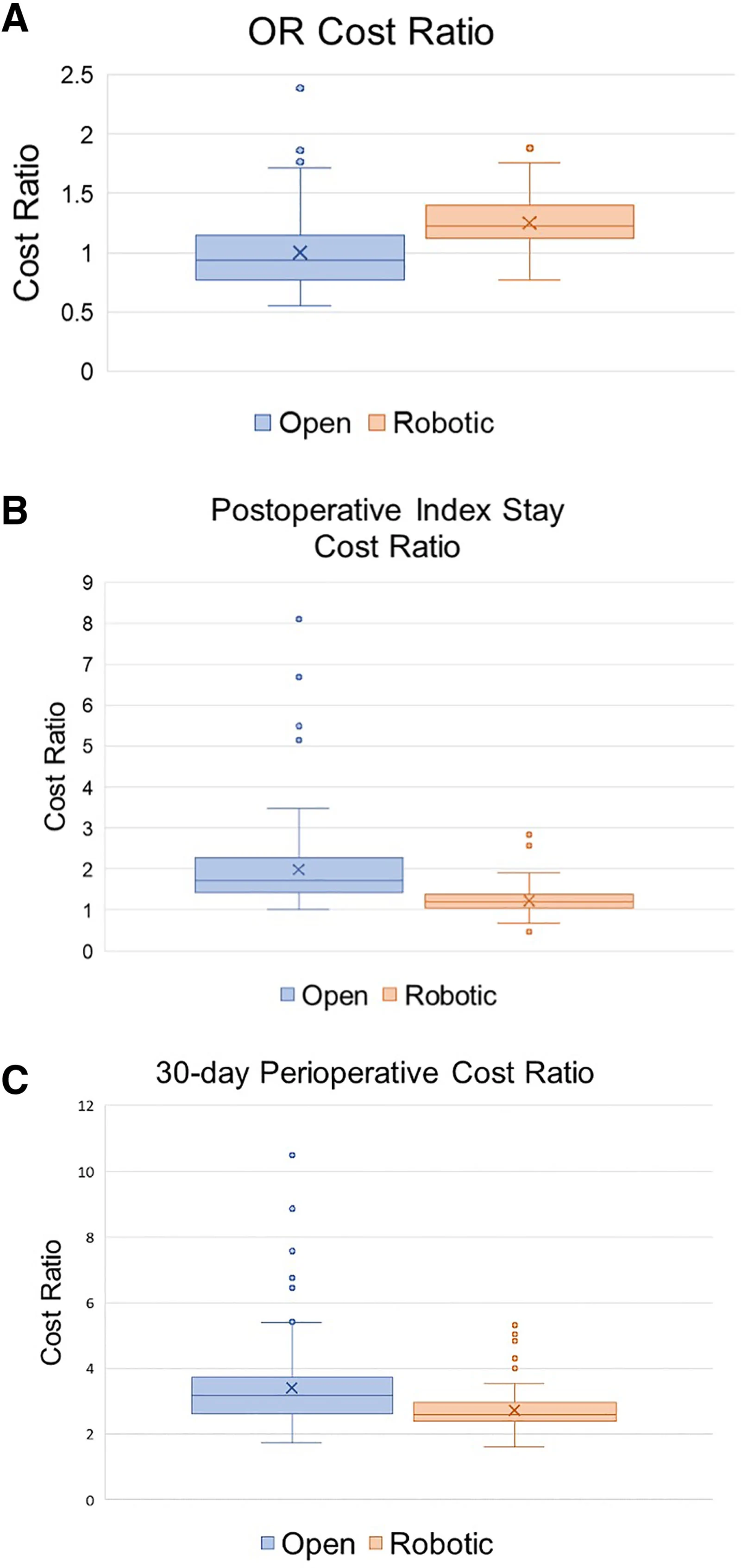
The (A) intraoperative (total operating room + anesthesia), (B) postoperative index stay, and (C) total 30-day perioperative cost ratios are shown according to surgical approach.

Costs are compared between ODP and RDP in Table 2 and Figure 1A–C. RDP had a higher mean intraoperative cost ratio than ODP (1.25 vs 1.00; *P* < 0.001), primarily due to the use of the robotic platform and instruments. However, RDP was associated with a lower mean postoperative inpatient care cost ratio (1.23 vs 1.99; *P* < 0.001). Exploratory analyses of the components of postoperative inpatient care costs revealed that this difference was mainly driven by lower costs for transfusions (0.09 vs 0.48), pharmacy (0.25 vs 0.51), regular inpatient room/board (0.23 vs 0.42), intermediate room care (0.28 vs 0.37), laboratory/pathology services (0.12 vs 0.18), and radiology imaging (0.08 vs 0.12) for RDP versus ODP, respectively, while costs were similar in intensive care unit (0.36 vs 0.38), physical/occupational/speech therapy (0.03 vs 0.05), recovery room (0.12 vs 0.12), and respiratory services (0.04 vs 0.03) for RDP versus ODP, respectively. As a result, the mean index stay cost, which includes the intraoperative cost and postoperative inpatient care, was significantly lower for RDP (2.48 vs 2.99; *P* < 0.001) than for ODP. The mean readmission cost ratio (0.07 vs 0.18; *P* = 0.088), mean outpatient cost ratio (0.19 vs 0.23; *P* = 0.36), and mean postdischarge total cost ratio (0.25 vs 0.42; *P* = 0.14) were similar between groups. Overall, the mean 30-day total perioperative cost was approximately 20% lower for RDP (2.73) than for ODP (3.41; *P* < 0.001). On multivariable linear regression, the robotic approach for distal pancreatectomy remained independently associated with lower 30-day costs (β −0.72, 95% CI −1.02 to −0.43; *P* < 0.001) (Supplemental Table 1, https://links.lww.com/AOSO/A630).

**TABLE 2.** Intraoperative, Inpatient, and Outpatient Cost Ratio According To Surgical Approach

		Approach		
Cost Ratio	Overall (N = 202)	Open (N = 101)	Robotic (N = 101)	*P* *
Index stay (intraoperative + postoperative inpatient care)	2.73 (2.26–2.99)	2.99 (2.32–3.24)	2.48 (2.16–2.65)	<0.001
Intraoperative (OR + anesthesia)	1.12 (0.90–1.29)	1.00 (0.77–1.14)	1.25 (1.12–1.38)	<0.001
Postoperative inpatient care	1.61 (1.15–1.75)	1.99 (1.42–2.23)	1.23 (1.04–1.38)	<0.001
Postdischarge total	0.33 (0.06–0.29)	0.42 (0.07–0.33)	0.25 (0.05–0.25)	0.14
Readmission	0.12 (0.00–0.00)	0.18 (0.00–0.00)	0.07 (0.00–0.00)	0.088
Outpatient	0.21 (0.06–0.22)	0.23 (0.06–0.22)	0.19 (0.05–0.22)	0.36
30-day total perioperative	3.07 (2.42–3.37)	3.41 (2.61–3.70)	2.73 (2.39–2.98)	<0.001

All values are mean (interquartile range) unless otherwise indicated.

* Wilcoxon rank-sum test; Pearson chi-squared test; Fisher exact test.

OR indicates operating room.

The ODP cost demonstrated significantly greater variance in postoperative inpatient care (*F* = 14.9, *P* < 0.001) and 30-day total costs (*F* = 13.33, *P* < 0.001), as visually demonstrated by the narrower distribution of RDP costs in Figure 1A–C.

### Other Surgical Outcomes

Surgical outcomes of the matched cohorts are summarized in Table 3. Compared with ODP, RDP was associated with a shorter median length of stay (3 days vs 5 days; *P* < 0.001), lower median EBL (50 mL vs 200 mL; *P* < 0.001), and lower incidence of chyle leak (1% vs 8%; *P* = 0.035). The rates of major complications (Accordion grade ≥3: 10% vs 17%; *P* = 0.15), including pulmonary embolisms (0% vs 3%; *P* = 0.25), infectious (11% vs 18%; *P* = 0.16), pulmonary complications (4% vs 5%; *P* > 0.99), and cardiac complications (6% vs 4%; *P* = 0.52), were comparable between RDP and ODP. There were similar median operative times (266 minutes vs 268 minutes; *P* = 0.95) and 90-day readmission rates (17% vs 17%; *P >* 0.99).

**TABLE 3.** Patients’ 90-Day Outcomes According to Surgical Approach

		Approach		
Characteristic	Overall (N = 202)	Open (N = 101)	Robotic (N = 101)	*P* *
Intraoperative factors				
Median EBL, mL (IQR)	100 (50–200)	200 (100–300)	50 (25–100)	<0.001
Median length of surgery, minutes (IQR)	268 (215–318)	268 (211–317)	266 (220–318)	0.95
Postoperative complications				
Accordion grade ≥3	27 (13)	17 (17)	10 (10)	0.15
Infectious complications	29 (14)	18 (18)	11 (11)	0.16
Abscess	5 (3)	4 (4)	1 (1)	0.37
Line infection	1 (1)	1 (1)	0 (0)	>0.99
Urinary tract infection	9 (5)	3 (3)	6 (6)	0.50
Wound infection	7 (4)	6 (6)	1 (1)	0.12
Colitis	2 (1)	2 (2)	0 (0)	0.50
Sepsis	1 (1)	0 (0)	1 (1)	>0.99
Pulmonary complication	9 (5)	5 (5)	4 (4)	>0.99
Cardiac complication	10 (5)	4 (4)	6 (6)	0.52
POPF				
Biochemical leak	54 (27)	20 (20)	34 (34)	0.026
Grade B POPF	34 (17)	21 (21)	13 (13)	0.13
DGE				0.62
Grade A	4 (2)	3 (3)	1 (1)	
Grade B	1 (1)	0 (0)	1 (1)	
None	197 (98)	98 (97)	99 (98)	
Postoperative transfusion	8 (4)	6 (6)	2 (2)	0.28
Chyle leak	9 (5)	8 (8)	1 (1)	0.035
Pulmonary embolism	3 (2)	3 (3)	0 (0)	0.25
Deep vein thrombosis	2 (1)	0 (0)	2 (2)	0.50
Median length of stay, days (IQR)	4 (3–5)	5 (4–6)	3 (3–4)	<0.001
Readmission within 90 days	34 (17)	17 (17)	17 (17)	>0.99
Death within 90 days	1 (1)	1 (1)	0 (0)	>0.99

All values are n (%) unless otherwise indicated.

* Wilcoxon rank-sum test; Pearson chi-squared test; Fisher exact test.

DGE indicates delayed gastric emptying; EBL, estimated blood loss; IQR, interquartile range; POPF, postoperative pancreatic fistula.

Notably, RDP had higher rates of biochemical leaks (34% vs 20%; *P* = 0.026), but grade B postoperative pancreatic fistula rates were similar (13% vs 21%; *P* = 0.13), as were rates of pancreatic fistulas requiring radiologic interventions (7% vs 11%; *P =* 0.32). One 90-day postoperative death occurred in the ODP group, but none occurred in the RDP group.

RDP and ODP patients had similar median WBC counts preoperatively and on postoperative day (POD) 0 to 2 (Supplemental Figure 1, https://links.lww.com/AOSO/A633). RDP patients had higher median WBC counts on POD3 (15.7 K/µL vs 13.7 K/µL; *P* = 0.003). However, the WBC counts were similar for the RDP and ODP patients at the first postoperative visit (10.9 K/µL vs 10.7 K/µL; *P* = 0.93). RDP patients had higher median DFA levels on POD1 (2286 U/L vs 1129 U/L; *P* = 0.003) but similar levels to those of ODP patients on POD3 (232 U/L vs 197 U/L; *P* = 0.63). DFA levels were unable to be tested in 2 RDP patients on POD1 due to a small volume of output, and 30 (15%) patients (23 RDP vs 7 ODP; *P* = 0.002) did not have DFA levels from POD3 available because the drain had already been removed or the output volume was too low. A total of 61 (30%) patients had their drains removed on or before POD3 (35% RDP vs 26% ODP; *P* = 0.17).

### Opioid Use Comparisons

Table 4 summarizes the differences in opioid use between RDP and ODP patients during perioperative care. The median intraoperative OME was similar (25 mg vs 25 mg) between the groups. However, RDP was associated with a lower median OME on POD0 (postoperative amount, 10 mg vs 15 mg), POD1 (12 mg vs 35 mg), POD2 (7 mg vs 23 mg), and POD3 (0 mg vs 10 mg), all with *P* < 0.001. Consequently, the cumulative median POD0-3 OME (36 mg vs 87 mg; *P* < 0.001) and the total inpatient OME (36 mg vs 111 mg; *P* < 0.001) were substantially lower for RDP.

**TABLE 4.** Intraoperative, Inpatient, and Discharge Oral Morphine Equivalent According to Surgical Approach

		Approach		
Characteristic	Overall (N = 202)	Open (N = 101)	Robotic (N = 101)	*P* *
Intraoperative OME, mg	25 (20–35)	25 (20–35)	25 (20–35)	0.75
POD0-3 total OME, mg	57 (24–109)	87 (46–163)	36 (15–68)	<0.001
Total inpatient OME, mg	59 (24–129)	111 (48–173)	36 (15–73)	<0.001
Discharge OME, mg	25 (0–75)	25 (0–100)	13 (0–60)	0.088

All values are median (interquartile range) unless otherwise indicated.

* Wilcoxon rank-sum test; Pearson chi-squared test; Fisher exact test.

mg indicates milligram; OME, oral morphine equivalents; POD, postoperative day.

Discharge OME prescriptions were similar between groups (median, RDP 13 mg vs ODP 25 mg, *P* = 0.088). Two patients in the ODP cohort received epidurals, removed on POD1 and POD2, respectively. No RDP patients received epidurals. Supplemental Figure 3a, https://links.lww.com/AOSO/A635 illustrates opioid consumption by POD for both RDP and ODP patients, along with the discharge OME prescriptions (Supplemental Figure 3b, https://links.lww.com/AOSO/A636).

## DISCUSSION

In this study, we compared the perioperative costs of RDP and ODP during the initial phase of our cancer center’s robotic pancreatectomy program using PSM. We also assessed 90-day surgical complications and postoperative opioid consumption. RDP was associated with lower 30-day total costs despite higher intraoperative costs, substantially reduced postoperative opioid use, shorter length of hospital stay, and otherwise similar 90-day outcomes. These findings support the safe implementation of our robotic pancreatectomy program at our center but also demonstrate the potential benefits of RDP even during surgeons’ early experience in a carefully built robotic program.

In this study, contrary to our expectations, we found that the 30-day total perioperative cost—including intraoperative, hospitalization, readmission, and outpatient care—was 20% lower for RDP than ODP. Although RDP incurred higher operating room costs due to the use of expensive robotic instruments, these costs were offset by substantially lower inpatient costs, largely attributable to shorter hospital stays. Additionally, we observed fewer outliers and reduced variability in total perioperative costs within the RDP cohort. This decreased variability may suggest that, despite similar complication rates, RDP may help mitigate the downstream financial impact of complications and facilitate more consistent adherence to standardized postoperative care pathways.

While our findings support broader adoption of robotic programs to offset the increasing healthcare costs in the US, caution is needed when applying these results internationally. In the US—where each inpatient day costs $2800 to $4100 USD^29,30^—shorter stays substantially offset operative expenses associated with robotic surgery. However, in countries with national insurance systems and much lower hospitalization costs (often less than one-third of US costs for inpatient care),^31,32^ these savings may not outweigh the higher operative costs. In these countries, the implementation and reimbursement of robotic surgery have been regulated by governments focused on cost-effectiveness,^33^ whereas in the US, the adoption of robotic surgery has been largely driven by market fundamentalism.^34,35^ Further studies are needed to evaluate perioperative costs in the context of these country-specific differences.

This study demonstrated similar 90-day outcomes for RDP and ODP. Both approaches had similar major complication rates (defined as Accordion grade ≥3); clinically relevant postoperative pancreatic fistula (CR-POPF); pulmonary, cardiac, infectious, and thromboembolic complications; postoperative transfusion rates; and 90-day readmission and mortality rates.

These findings align with prior studies that reported no significant differences in CR-POPF, hemorrhage, or 30-day mortality between the 2 approaches.^36,37^ RDP had a shorter median length of stay (3 vs 5 days) and a reduction in EBL, consistent with findings from earlier literature.^38,39^ Whereas previous studies reported longer operative times for RDP than ODP,^9,10^ our study found similar operative times between the 2 approaches, suggesting that RDP can be performed efficiently—even during the learning curve—when implemented within a structured program.

RDP was associated with substantially reduced inpatient opioid use, a trend seen in other robotic operations.^16,40^ Although the mechanisms behind the reduced opioid use in robotic surgery are not fully understood, factors associated with robotic surgery, such as smaller incisions, more direct dissection, and reduced peritoneal trauma, may lead to faster gastrointestinal recovery and contribute to reduced pain and a lower need for opioids.^41,42^ This opioid-sparing effect may support improved recovery, as lower opioid use in the first three PODs has been associated with reduced CR-POPF rates.^43^ In the context of the opioid epidemic, this highlights robotic surgery’s role in promoting value-based care.^34^ Despite differences in inpatient use, discharge opioid prescriptions were similar. On the other hand, our institutional efforts to reduce opioid use—by the iterative implementation of risk-stratified pancreatectomy pathways with a standardized discharge prescription algorithm based on the final 24 hours of inpatient use—contributed to minimizing opioid prescriptions regardless of the surgical approach.^44,45^

With our fully implemented RDP program, in which about half of distal pancreatectomies have recently been performed robotically, this study suggests that a specific pathway is needed to guide postoperative care after RDP. While POD1 and 3 DFA levels currently guide drain removal,^17,18,46–48^ our data suggest that RDP follows a different DFA trajectory than ODP, with higher POD1 DFA and more biochemical leaks but similar CR-POPF rates. This may reflect differences in peritoneal inflammation and ascites between approaches; these differences affect drain fluid concentration^49,50^ and support the need for different DFA thresholds in RDP. Drains were more often removed on or before POD3 in the RDP group (35% vs 26%) without increased rates of CR-POPF or readmission, suggesting that the current DFA thresholds may be overly conservative for RDP. We also observed higher WBC counts on POD3 and at discharge in the RDP group, although the median levels normalized by follow-up, and there were no differences in infectious complications or readmissions. The cause of this difference remains unclear.

Our study has several limitations. First, its retrospective, single-institution design introduces selection bias and limits generalizability. Most notably, the involvement of multiple surgeons with varying experience levels and thresholds for selecting patients for RDP introduced surgeon-related bias and limited generalizability. As the study period included the implementation phase of the RDP program, approximately two-thirds of RDP cases involved the lead surgeon (NI) as a primary or co-surgeon, whereas ODP cases were rarely performed by him (Supplemental Table 2, https://links.lww.com/AOSO/A631). Although we used PSM to minimize confounding and balance patient and disease characteristics, unmeasured or unknown confounders may still have influenced the outcomes. In addition, inherent differences in the extent and technical complexity of the operations mean the 2 groups cannot be considered fully equivalent. Additionally, more cases from the ODP cohort were performed in early 2018, whereas the RDP program was initiated in late 2018, introducing some imbalance in the year of surgery between the 2 cohorts (Supplemental Table 3, https://links.lww.com/AOSO/A632). However, the cost trend for individual cases over time did not show substantial changes (Supplemental Figure 2, https://links.lww.com/AOSO/A634). That said, the primary objective of this study was to evaluate the safety and cost-effectiveness of a strategically and collaboratively implemented RDP program. In this context, comparing 2 cohorts managed by the same surgical team remains both meaningful and valid. Furthermore, the cost analysis was limited to 30-day hospital billing data and did not include physician fees, readmissions at outside institutions, or long-term or indirect costs such as those related to postoperative chemotherapy. Therefore, while we observed reduced perioperative costs with RDP, the total healthcare costs may have been underestimated. Finally, we did not assess long-term outcomes, patient-reported measures, or broader economic impacts, including indirect costs like lost productivity. Importantly, longer-term complications such as incisional hernia following laparotomy were not captured in this analysis. Given that incisional hernia rates may be higher following midline incision compared to Pfannenstiel incision, the downstream costs associated with these complications could further influence the long-term cost comparison between open and minimally invasive approaches.

## CONCLUSIONS

This study demonstrated that RDP has similar short-term outcomes to those of ODP and significantly reduced opioid use and lower 30-day perioperative costs. These findings support the safe and cost-effective adoption of robotic pancreatectomy, particularly within structured programs that provide appropriate training and mentorship. Our results suggest that a tailored, RDP-specific postoperative pathway—designed to accommodate distinct inflammatory and biochemical profiles—may further enhance recovery. Future research should aim to optimize these pathways, refine criteria for drain removal, and assess the long-term outcomes and cost-effectiveness of RDP to better define its role in value-based surgery.

## ACKNOWLEDGMENTS

We thank Madison Semro, Associate Scientific Editor, and Sarah Bronson, Scientific Editor, in the Research Medical Library at The University of Texas MD Anderson Cancer Center for editing this article.

## Supplementary Material



## References

[1] Caba Molina D Lambreton F Arrangoiz Majul R. Trends in robotic pancreaticoduodenectomy and distal pancreatectomy. J Laparoendosc Adv Surg Tech A. 2019;29:147–151. 10.1089/lap.2018.0421 30222522

[2] Royall NA Walsh RM. Robotic distal pancreatectomy and splenectomy: rationale and technical considerations. J Vis Surg. 2017;3:135. 10.21037/jovs.2017.08.01 PMC5639012 29078695

[3] de Rooij T van Hilst J van Santvoort H; Dutch Pancreatic Cancer Group. Minimally invasive versus open distal pancreatectomy (LEOPARD): a multicenter patient-blinded randomized controlled trial. Ann Surg. 2019;269:2–9. 10.1097/SLA.0000000000002979 30080726

[4] Al Abbas AI Zeh Iii HJ Polanco PM. State of the art robotic distal pancreatectomy: a review of the literature. Updates Surg. 2021;73:881–891. 10.1007/s13304-021-01070-y 34050901

[5] Müller PC Breuer E Nickel F. Robotic distal pancreatectomy: a novel standard of care? Benchmark values for surgical outcomes from 16 international expert centers. Ann Surg. 2023;278:253–259. 10.1097/SLA.0000000000005601 35861061

[6] Palep JH. Robots assisted minimally invasive surgery. J Minim Access Surg. 2009;5:1–7. 10.4103/0972-9941.51313 PMC2699074 19547687

[7] Kamarajah SK Sutandi N Robinson SR. Robotic versus conventional laparoscopic distal pancreatic resection: a systematic review and meta-analysis. HPB (Oxford). 2019;21:1107–1118. 10.1016/j.hpb.2019.02.020 30962137

[8] Li P Zhang H Chen L. Robotic versus laparoscopic distal pancreatectomy on perioperative outcomes: a systematic review and meta-analysis. Updates Surg. 2023;75:7–21. 10.1007/s13304-022-01413-3 PMC9834369 36378464

[9] Al Abbas AI Wang C Hamad AB. Mentorship and formal robotic proficiency skills curriculum improve subsequent generations’ learning curve for the robotic distal pancreatectomy. HPB (Oxford). 2021;23:1849–1855. 10.1016/j.hpb.2021.04.022 34059420

[10] Chen C Hu J Yang H. Is robotic distal pancreatectomy beter than laparoscopic distal pancreatectomy after the learning curve? A systematic review and meta-analysis. Front Oncol. 2022;12:954227. 10.3389/fonc.2022.954227 PMC9465417 36106111

[11] Hirata Y Prakash L Maxwell J. Institutional learning curve and factors of prolonged operation time of robotic distal pancreatectomy: an analysis of an initial 117 cases. Ann Gastroenterol Surg. 2025;9:861–869. 10.1002/ags3.70005 PMC12211093 40607303

[12] Hirata Y Lyu HG Azimuddin AM. Cost analysis for robotic and open gastrectomy. Ann Surg Open. 2024;5:e396. 10.1097/AS9.0000000000000396 PMC11175903 38883961

[13] Ma LW Dominguez-Rosado I Gennarelli RL. The cost of postoperative pancreatic fistula versus the cost of pasireotide: results from a prospective randomized trial. Ann Surg. 2017;265:11–16. 10.1097/SLA.0000000000001892 PMC5182113 27429029

[14] Partelli S Ricci C Cinelli L. Evaluation of cost-effectiveness among open, laparoscopic and robotic distal pancreatectomy: a systematic review and meta-analysis. Am J Surg. 2021;222:513–520. 10.1016/j.amjsurg.2021.03.066 33853724

[15] Soomro NA Hashimoto DA Porteous AJ. Systematic review of learning curves in robot-assisted surgery. BJS Open. 2020;4:27–44. 10.1002/bjs5.50235 PMC6996634 32011823

[16] MacQueen IT Milky G Shih IF. New persistent opioid use following robotic-assisted, laparoscopic and open surgery inguinal hernia repair. Surg Endosc. 2024;38:5153–5159. 10.1007/s00464-024-11040-1 PMC11362387 39039294

[17] Denbo JW Bruno M Dewhurst W. Risk-stratified clinical pathways decrease the duration of hospitalization and costs of perioperative care after pancreatectomy. Surgery. 2018;164:424–431. 10.1016/j.surg.2018.04.014 PMC7265789 29807648

[18] Boyev A Jain AJ Newhook TE. Opioid-free discharge after pancreatic resection through a learning health system paradigm. JAMA Surg. 2023;158:e234154. 10.1001/jamasurg.2023.4154 PMC10483385 37672236

[19] Newhook TE Vega EA Vreeland TJ. Early postoperative drain fluid amylase in risk-stratified patients promotes tailored post-pancreatectomy drain management and potential for accelerated discharge. Surgery. 2020;167:442–447. 10.1016/j.surg.2019.09.015 PMC7288221 31727324

[20] Ikoma N Lee JE Katz MHG. ASO author reflections: accelerating the learning curve of robotic pancreatectomy and gastrectomy through a composite robotic foregut surgical oncology program. Ann Surg Oncol. 2022;29:286–287. 10.1245/s10434-021-10826-0 34595667

[21] Schwarz L Bruno M Parker NH. Active surveillance for adverse events within 90 days: the standard for reporting surgical outcomes after pancreatectomy. Ann Surg Oncol. 2015;22:3522–3529. 10.1245/s10434-015-4437-z PMC12047848 25694246

[22] Strasberg SM Linehan DC Hawkins WG. The Accordion severity grading system of surgical complications. Ann Surg. 2009;250:177–186. 10.1097/SLA.0b013e3181afde41 19638919

[23] Wente MN Veit JA Bassi C. Postpancreatectomy hemorrhage (PPH): an International Study Group Of Pancreatic Surgery (ISGPS) definition. Surgery. 2007;142:20–25. 10.1016/j.surg.2007.02.001 17629996

[24] Wente MN Bassi C Dervenis C. Delayed gastric emptying (DGE) after pancreatic surgery: a suggested definition by the International Study Group of Pancreatic Surgery (ISGPS). Surgery. 2007;142:761–768. 10.1016/j.surg.2007.05.005 17981197

[25] Bassi C Marchegiani G Dervenis C; International Study Group on Pancreatic Surgery (ISGPS). The 2016 update of the internafonal study group (ISGPS) definition and grading of postoperative pancreatic fistula: 11 years after. Surgery. 2017;161:584–591. 10.1016/j.surg.2016.11.014 28040257

[26] Kaplan RS Witkowski M Abboh M. Using time-driven activity-based costing to identify value improvement opportunities in healthcare. J Healthc Manag. 2014;59:399–412. 25647962

[27] Wih RG Hirata Y Prakash LR. Comparative analysis of opioid use between robotic and open pancreaticoduodenectomy. J Hepatobiliary Pancreat Sci. 2023;30:523–531. 10.1002/jhbp.1216 PMC9823147 35796581

[28] Hirata Y Gohumukkala V Ajith J. Laparoscopic transverse abdominis plane block: how I do it and a cost efficiency analysis. Langenbecks Arch Surg. 2023;409:16. 10.1007/s00423-023-03210-x 38147123

[29] Birkmeyer JD Gust C Dimick JB. Hospital quality and the cost of inpatient surgery in the United States. Ann Surg. 2012;255:1–5. 10.1097/SLA.0b013e3182402c17 PMC3249383 22156928

[30] Milliken M. Hospital and surgery costs. 2023. Available at: hhps://www.debt.org/medical/hospital-surgery-costs/. Accessed January 17, 2025.

[31] Figueroa JF Papanicolas I Riley K. International comparison of health spending and utilization among people with complex multimorbidity. Health Serv Res. 2021;56(Suppl 3):1317–1334. 10.1111/1475-6773.13708 PMC8579210 34350586

[32] Emma Wagner SR Cox C. What drives health spending in the U.S. compared to other countries? 2024. Available at: hhps://www.healthsystemtracker.org/brief/what-drives-health-spending-in-the-u-s-compared-to-other-countries/#Healthcare%20spending%20per%20capita,%20by%20spending%20category,%202021. Accessed January 17, 2025.

[33] Nishigori T Ichihara N Obama K. Prevalence and safety of robotic surgery for gastrointestinal malignant tumors in Japan. Ann Gastroenterol Surg. 2022;6:746–752. 10.1002/ags3.12579 PMC9628217 36338596

[34] Ikoma N. What defines the “value” of robotic surgery for patients with gastrointestinal cancers? Perspectives from a U.S. cancer center. Ann Gastroenterol Surg. 2024;8:566–579. 10.1002/ags3.12792 PMC11216793 38957558

[35] Kiyama T Tajiri T Yoshiyuki T. Implementation of the diagnosis procedure combination in specific-function hospitals. J Nippon Med Sch. 2004;71:217–220. 10.1272/jnms.71.217 15226615

[36] Zhou JY Xin C Mou YP. Robotic versus laparoscopic distal pancreatectomy: a meta-analysis of short-term outcomes. PLoS One. 2016;11:e0151189. 10.1371/journal.pone.0151189 PMC4790929 26974961

[37] Guerrini GP Laureha A Belluco C. Robotic versus laparoscopic distal pancreatectomy: an up-to-date meta-analysis. BMC Surg. 2017;17:105. 10.1186/s12893-017-0301-3 PMC5680787 29121885

[38] Gavriilidis P Roberts KJ Sutcliﬀe RP. Comparison of robotic vs laparoscopic vs open distal pancreatectomy. A systematic review and network meta-analysis. HPB (Oxford). 2019;21:1268–1276. 10.1016/j.hpb.2019.04.010 31080086

[39] Magistri P Boggi U Esposito A. Robotic vs open distal pancreatectomy: a multi-institutional matched comparison analysis. J Hepatobiliary Pancreat Sci. 2021;28:1098–1106. 10.1002/jhbp.881 33314791

[40] Saleh T Ford J Kindel T. Inpatient opioid use and pain control after robotic versus laparoscopic sleeve gastrectomy. Surgery. 2024;175:599–604. 10.1016/j.surg.2023.08.064 37981549

[41] Talwar R Joshi SS. Minimizing opioid consumption following robotic surgery. Transl Androl Urol. 2021;10:2289–2296. 10.21037/tau.2019.08.11 PMC8185686 34159111

[42] Woldu SL Weinberg AC Bergman A. Pain and analgesic use after robot-assisted radical prostatectomy. J Endourol. 2014;28:544–548. 10.1089/end.2013.0783 24400824

[43] Boyev A Prakash LR Chiang YJ. Postoperative opioid use is associated with increased rates of grade B/C pancreatic fistula after distal pancreatectomy. J Gastrointest Surg. 2023;27:2135–2144. 10.1007/s11605-023-05751-4 37468733

[44] DiPeri TP Newhook TE Day RW; PROMOTE Consortium. A prospective feasibility study evaluating the 5×-mulfplier to standardize discharge prescriptions in cancer surgery patients. Surg Open Sci. 2022;9:51–57. 10.1016/j.sopen.2022.04.004 PMC9161107 35663797

[45] DiPeri TP Newhook TE Tran Cao HS. Opioid discharge prescriptions after inpatient surgery: risks of rebound refills by length of stay. J Surg Res. 2022;278:111–118. 10.1016/j.jss.2022.04.057 35597025

[46] Arango NP Prakash LR Chiang YJ. Risk-stratified pancreatectomy clinical pathway implementafon and delayed gastric emptying. J Gastrointest Surg. 2021;25:2221–2230. 10.1007/s11605-020-04877-z 33236322

[47] Newton AD Newhook TE Bruno ML. Iterative changes in risk-stratified pancreatectomy clinical pathways and accelerated discharge after pancreaticoduodenectomy. J Gastrointest Surg. 2022;26:1054–1062. 10.1007/s11605-021-05235-3 35023033

[48] Ayabe RI Prakash LR Bruno ML. Differential gains in surgical outcomes for high-risk vs low-risk pancreaticoduodenectomy with successive refinements of risk-stratified care pathways. J Am Coll Surg. 2023;237:4–12. 10.1097/XCS.0000000000000652 36786469

[49] Giulianou PC Bianco FM Daskalaki D. Robotic liver surgery: technical aspects and review of the literature. Hepatobiliary Surg Nutr. 2016;5:311–321. 10.21037/hbsn.2015.10.05 PMC4960413 27500143

[50] Yoon YI Kim KH Kang SH. Pure laparoscopic versus open right hepatectomy for hepatocellular carcinoma in patients with cirrhosis: a propensity score matched analysis. Ann Surg. 2017;265:856–863. 10.1097/SLA.0000000000002072 27849661

