# Supplementary material for: Short-Term Outcomes and Cost of Robotic Versus Open Distal Pancreatectomy During the Initial Phase of a Robotic Pancreatectomy Program
Source: Ann Surg Open. 2026 Jul 2;7(3):e687. doi: 10.1097/AS9.0000000000000687 (PMC13596980; doi:10.1097/AS9.0000000000000687)
Supplement: Supplementary file 1 [file as9-7-e687-s001.pdf]

Supplemental Table 1: Multivariable linear regression analysis evaluating factors associated with 30-day total cost after distal pancreatectomy

Multivariable Linear Regression			
Variable	Beta	95% CI	p-value
Robotic Approach (reference: Open)	-0.72	(-1.02, -0.43)	<0.001
BMI	0.03	(0, 0.05)	0.044
Indication: Other (reference: Cyst)	-0.32	(-0.91, 0.27)	0.283
Indication: PDAC (reference: Cyst)	-0.29	(-0.68, 0.11)	0.150
Indication: PNET (reference: Cyst)	-0.31	(-0.7, 0.09)	0.125

Beta coefficients represent the estimated change in log-transformed cost associated with each variable.

CI, confidence interval; BMI, body mass index; PDAC, pancreatic ductal adenocarcinoma; PNET, pancreatic neuroendocrine tumor.
