# Supplementary material for: Short-Term Outcomes and Cost of Robotic Versus Open Distal Pancreatectomy During the Initial Phase of a Robotic Pancreatectomy Program
Source: Ann Surg Open. 2026 Jul 2;7(3):e687. doi: 10.1097/AS9.0000000000000687 (PMC13596980; doi:10.1097/AS9.0000000000000687)
Supplement: Supplementary file 2 [file as9-7-e687-s002.pdf]

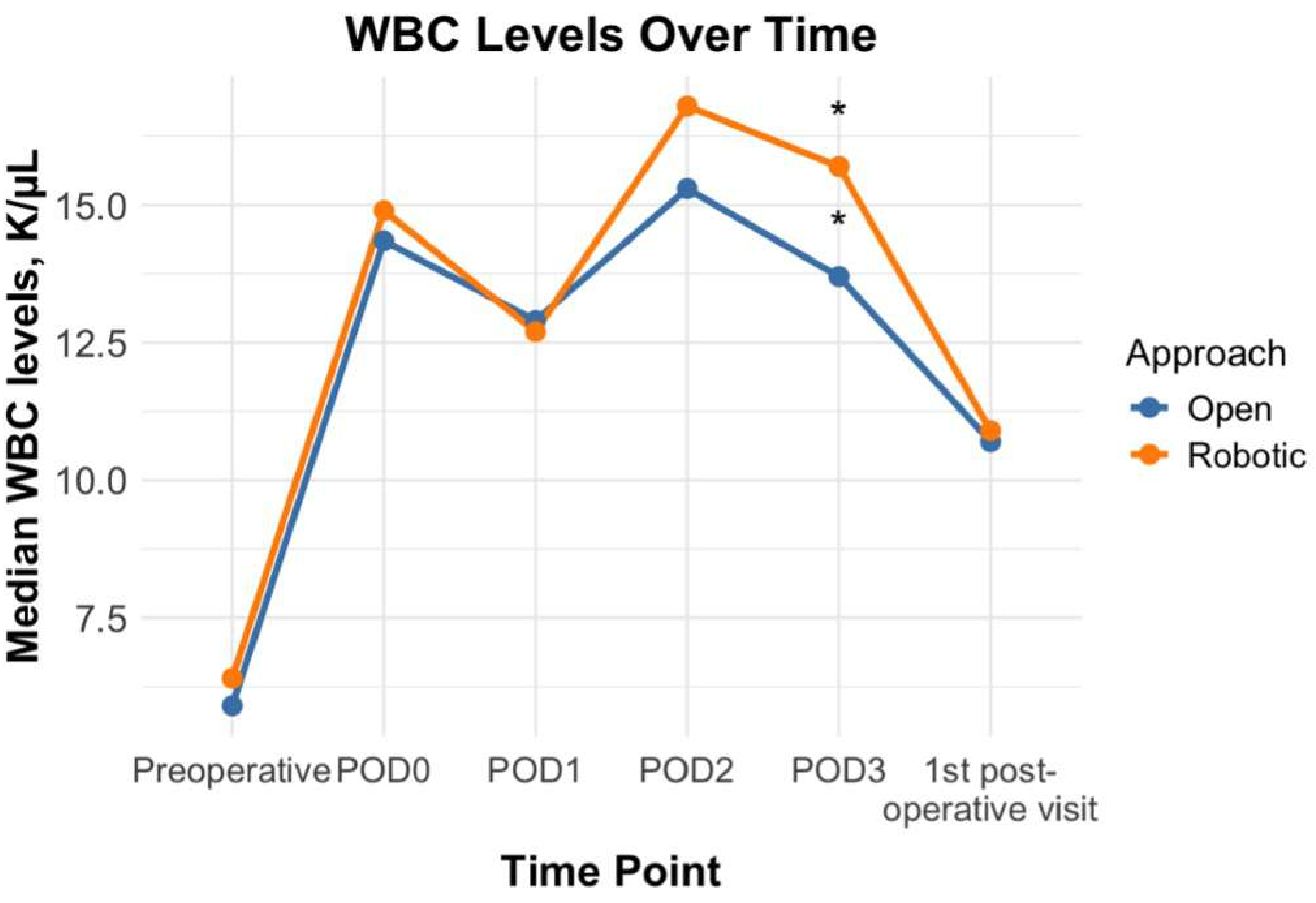

WBC Levels Over Time
Median WBC levels, K/μL
15.0
12.5
10.0
7.5
*
*
Preoperative
POD0
POD1
POD2
POD3
1st post-operative visit
Time Point
Approach
Open
Robotic
