# Supplementary material for: Short-Term Outcomes and Cost of Robotic Versus Open Distal Pancreatectomy During the Initial Phase of a Robotic Pancreatectomy Program
Source: Ann Surg Open. 2026 Jul 2;7(3):e687. doi: 10.1097/AS9.0000000000000687 (PMC13596980; doi:10.1097/AS9.0000000000000687)
Supplement: Supplementary file 3 [file as9-7-e687-s003.pdf]

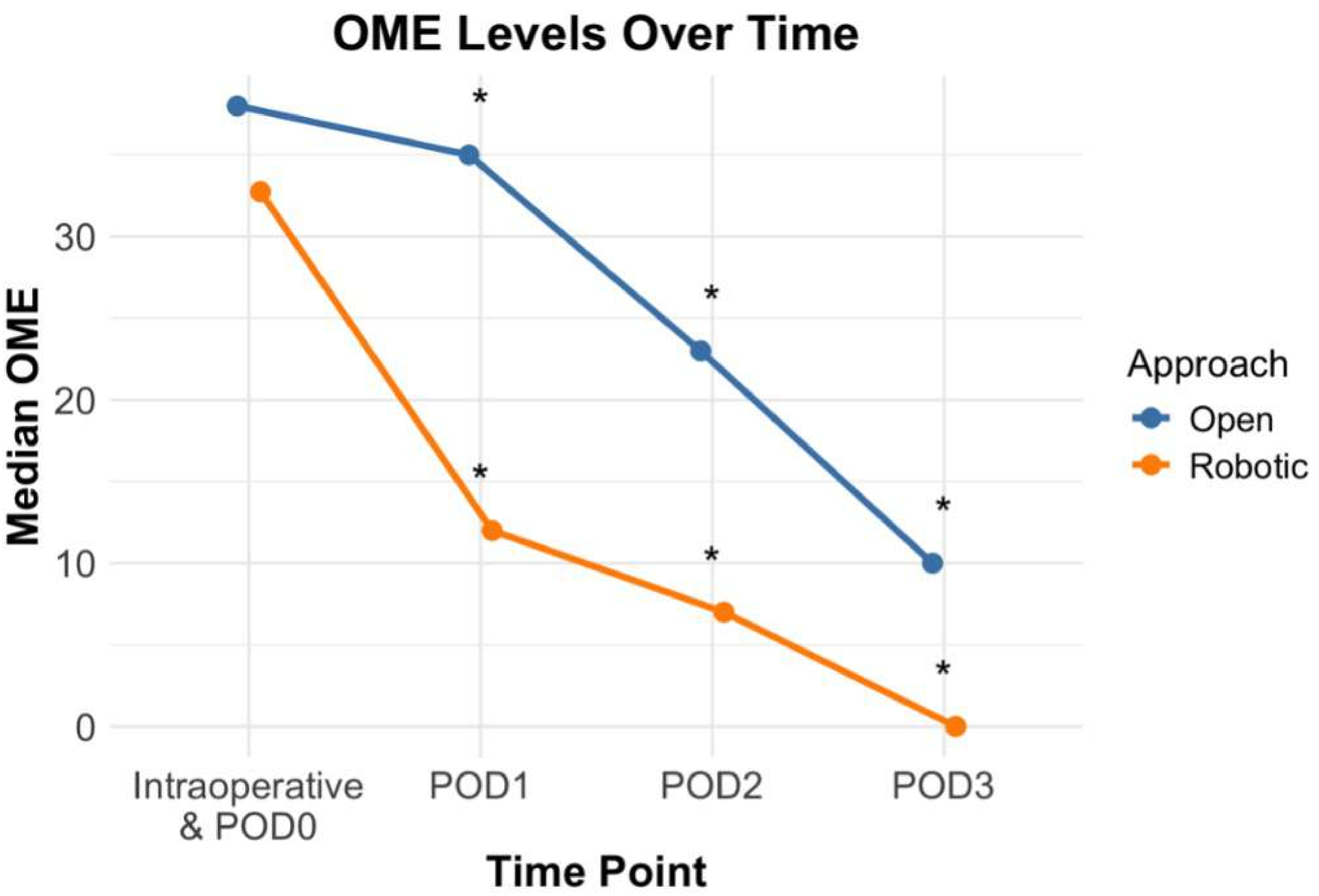
OME Levels Over Time
Median OME
30
20
10
0
*
*
*
*
*
*
Intraoperative & POD0
POD1
POD2
POD3
Time Point
Approach
Open
Robotic
