# Supplementary material for: Short-Term Outcomes and Cost of Robotic Versus Open Distal Pancreatectomy During the Initial Phase of a Robotic Pancreatectomy Program
Source: Ann Surg Open. 2026 Jul 2;7(3):e687. doi: 10.1097/AS9.0000000000000687 (PMC13596980; doi:10.1097/AS9.0000000000000687)
Supplement: Supplementary file 4 [file as9-7-e687-s004.pdf]

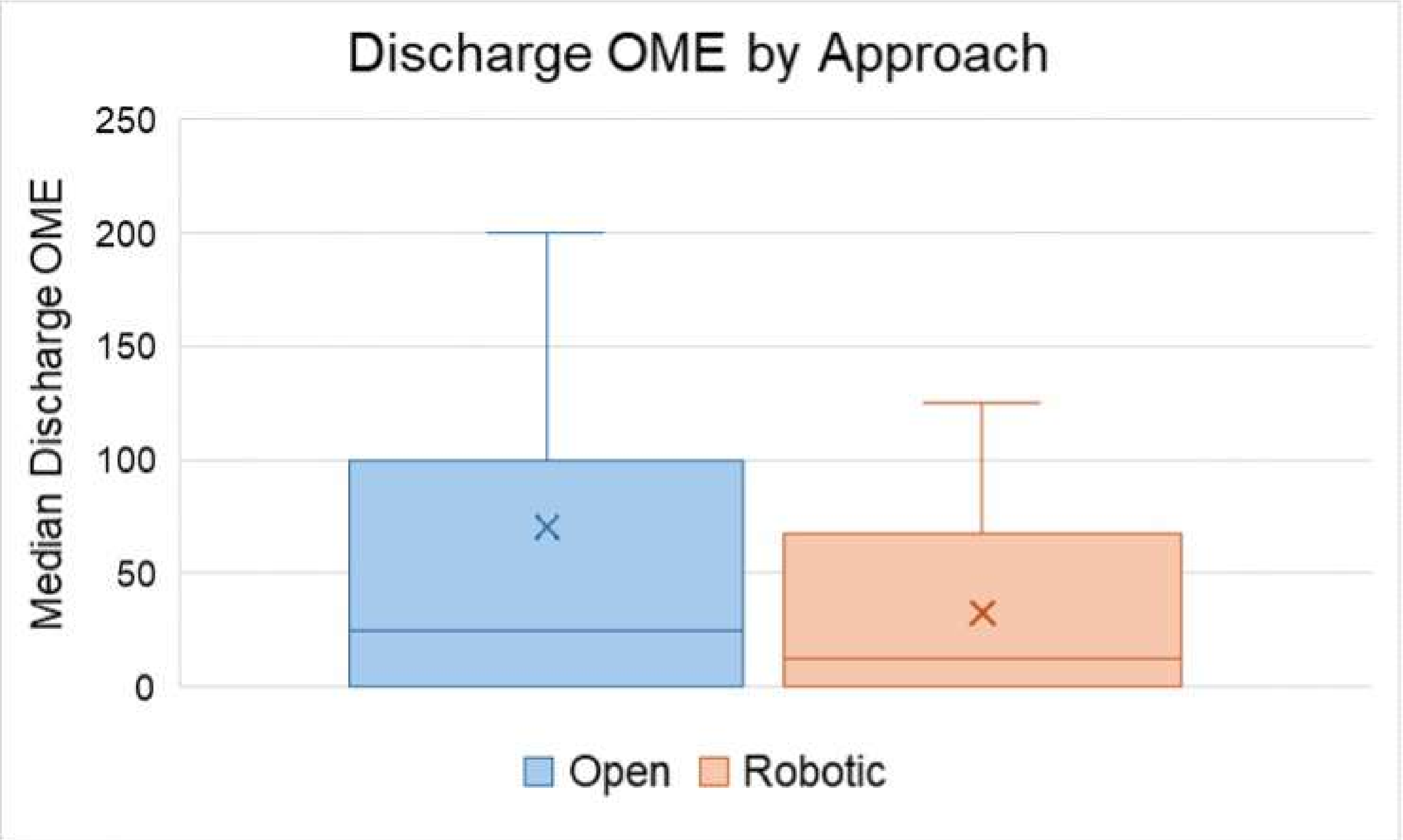
Discharge OME by Approach
250
200
150
100
50
0
Median Discharge OME
Open
Robotic
