# Supplementary material for: Short-Term Outcomes and Cost of Robotic Versus Open Distal Pancreatectomy During the Initial Phase of a Robotic Pancreatectomy Program
Source: Ann Surg Open. 2026 Jul 2;7(3):e687. doi: 10.1097/AS9.0000000000000687 (PMC13596980; doi:10.1097/AS9.0000000000000687)
Supplement: Supplementary file 5 [file as9-7-e687-s005.pdf]

Supplemental Table 2: Number of cases for surgeons and co-surgeons by approach.

	**Surgeon A**	**Surgeon B**	**Surgeon C**	**Surgeon D**	**Surgeon E**	**Surgeon F**	**Surgeon G**	**Total**
Open	0	42	22	5	16	7	9	101
Robotic	51	5	30	0	3	1	11	101
(multi-surgeon cases)	(13)	(5)	(8)	(0)	(3)	(1)	(6)	(36)
