# Supplementary material for: Short-Term Outcomes and Cost of Robotic Versus Open Distal Pancreatectomy During the Initial Phase of a Robotic Pancreatectomy Program
Source: Ann Surg Open. 2026 Jul 2;7(3):e687. doi: 10.1097/AS9.0000000000000687 (PMC13596980; doi:10.1097/AS9.0000000000000687)
Supplement: Supplementary file 6 [file as9-7-e687-s006.pdf]

Supplemental Table 3: Number of cases included after propensity score matching.

**Year**	**Open**	**Robotic**	**Total**
2018	25	1	26
2019	8	18	26
2020	11	9	20
2021	12	25	37
2022	19	19	38
2023	20	25	45
2024	6	4	10
